# Overlap between body composition abnormalities and sex-specific prognostication in decompensated cirrhosis

**DOI:** 10.3389/fnut.2025.1705226

**Published:** 2026-01-13

**Authors:** Jie Yang, Yan Song, Qing Liu, Chao Sun

**Affiliations:** 1 Department of Gastroenterology and Hepatology, Tianjin Medical University General Hospital, Tianjin, China; 2 Department of Gastroenterology, Tianjin Medical University General Hospital Airport Hospital, Tianjin, China; 3 Department of Gastroenterology, The First Affiliated Hospital of Xi’an Medical University, Xi’an, Shaanxi, China

**Keywords:** body composition, liver cirrhosis, myosteatosis, overlap, sarcopenia

## Abstract

**Purpose:**

We aimed to demonstrate distinct body composition (BC) profiles stratified by sex and clarify their joint effects on long-term mortality in a retrospective cohort of inpatients.

**Methods:**

Various BC parameters annotated on computed tomography (CT) images at the third lumbar vertebra were used to define sarcopenia, myosteatosis, low subcutaneous adiposity, and high visceral adiposity. These categories were constructed using sex-specific, outcome-based cutoffs in a prerequisite manner.

**Results:**

Among 519 patients hospitalized for acute decompensating episodes, the median age was 64.0 years, with a slight female predominance (51.6%). Among the female patients, high visceral adiposity was the most prevalent single BC abnormality (38.4%), while the most common overlapping phenotype was myosteatosis occurring concurrently with high visceral adiposity (9.7%). Among the male patients, high visceral adiposity also showed the highest prevalence (74.9%), while the most common overlapping phenotype was sarcopenia occurring concurrently with low subcutaneous adiposity (15.1%). Considering their jointly negative impact, the female patients experiencing three BC abnormalities had the lowest survival rate (33.3%, log-rank test: *p* = 0.0022). Still, this difference was only marginally significant in the male patients with three or more BC abnormalities (log-rank test: *p* = 0.068). Furthermore, overlapped BC abnormalities were associated with 722 and 331% higher risks, respectively, of 1-year all-cause mortality (*p* = 0.001) in the female patients relative to those with no BC abnormalities and those with an isolated BC abnormality. Lastly, our established nomogram integrated albumin, Model for End-Stage Liver Disease-Sodium (MELD-Na) score, and distinct overlapping BC abnormalities, demonstrating moderate accuracy, sufficient calibration, and clinical benefits for prognostication.

**Conclusion:**

In conclusion, sex-specific variations in BC profiles were observed among the patients with decompensated cirrhosis.

## Introduction

Liver cirrhosis represents the terminal stage of various chronic liver diseases and is a major cause of mortality, accounting for approximately 2.4% of deaths worldwide (1). Apart from aggravating hepatocellular carcinoma, cirrhosis can also present with a range of decompensating events, including variceal bleeding, ascites, hepatic encephalopathy, spontaneous bacterial peritonitis, and hepatorenal syndrome (2, 3). In this regard, the most commonly used and clinically adopted prognostic scoring systems—such as the Child–Pugh score and the Model for End-Stage Liver Disease (MELD) score—were developed several decades ago and have not undergone timely updates. However, accumulating evidence indicates a decrease in the predictive power of the MELD score, largely due to the evolving etiologies of cirrhosis (4–6). For instance, the epidemics of obesity and type 2 diabetes mellitus have driven a rapid increase in the prevalence of metabolic dysfunction-associated fatty liver disease; at the same time, the consumption of alcohol and alcohol-related liver disease are also increasing (7). Therefore, it is imperative to consider patients’ metabolic and nutritional status to more accurately predict healthcare outcomes and guide individualized therapeutic strategies. Accordingly, the assessment of body composition (BC) abnormalities provides an objective evaluation, and their negative impact on morbidity and mortality in the context of cirrhosis has been underscored in the existing literature.

BC refers to the quantitative distribution of major tissue compartments in the human body, primarily including skeletal muscle, subcutaneous adipose tissue (SAT), and visceral adipose tissue (VAT), all of which play critical roles in maintaining metabolic homeostasis and nutritional status. The presence of sarcopenia, myosteatosis, low subcutaneous adiposity, and high visceral adiposity—defined according to sex-specific cutoffs and linked to clinical outcomes in our previous report—was based on the respective skeletal muscle index (SMI), intramuscular adipose tissue content (IMAC), SATI, and VATI (8, 9). The specific categories pertinent to each BC abnormality are demonstrated in Supplementary Table S1.

It is highlighted that the nature of human BC is multifaceted, complex, and heterogeneous, particularly with respect to muscle and adipose tissues (10). More recently, pathological alterations of skeletal muscle—namely sarcopenia (loss of muscle quantity) and myosteatosis (impairment of muscle quality)—along with abnormalities in distinct adipose tissue compartments, including low subcutaneous adiposity, high visceral adiposity, and sarcopenic obesity, and their connection with prognostication have attracted increasing attention in the medical field (11–13). Notably, sex differences in BC have been increasingly recognized in clinical research. Female and male individuals inherently differ in hormone levels, metabolism, muscle morphology, and physical activity patterns, leading to distinct distributions of skeletal muscle, SAT, and VAT (14, 15). Although the association between a single BC abnormality and adverse outcomes has been well established, knowledge gaps do exist regarding the combined impact of multiple BC abnormalities in the context of cirrhosis (16). However, in the context of decompensated cirrhosis, how inherent sex differences influence the prevalence and combination patterns of BC abnormalities, as well as their joint impact on prognosis, remains poorly understood. Most previous studies have focused on single BC abnormalities without sex stratification or evaluation of the combined effects of multiple abnormalities, resulting in a critical knowledge gap in personalized prognostication and treatment for male and female patients with decompensated cirrhosis (16). Therefore, we hypothesized that the coexistence of BC abnormalities may have a superimposed effect on prognostication, with potential sex-specific variations in its clinical impact. The present study aimed to (1) demonstrate distinct BC profiles stratified by sex, (2) clarify their joint impact on long-term mortality, and (3) develop a predictive model incorporating both significant BC abnormalities and other independent risk factors in patients with decompensated cirrhosis.

## Methods

### Study population

A retrospective cohort study was carried out among consecutive patients hospitalized for cirrhosis-related acute decompensating events. Decompensation was defined as the presence of one or more of the following: Gastroesophageal variceal bleeding confirmed by endoscopic examination; ascites clinically evident on ultrasonography or physical examination and classified according to the International Ascites Club criteria; hepatic encephalopathy graded I–IV based on the West Haven Criteria; or jaundice, defined as a total bilirubin level of ≥51 μmol/L (17). In contrast, acute-on-chronic liver failure was defined by the presence of severe jaundice, indicated by a total bilirubin level of ≥85 μmol/L, and coagulation abnormalities, indicated by a prothrombin time–international normalized ratio (PT-INR) of ≥1.5, complicated by hepatic encephalopathy and/or ascites developing within 4 weeks in the context of cirrhosis or advanced chronic liver disease. The exclusion criteria were defined as follows: (1) age less than 18 years, (2) concomitant acute-on-chronic liver failure at index hospitalization, (3) hepatocellular carcinoma and/or other extrahepatic malignancies, and (4) absence of abdominal computed tomography (CT) images obtained within 3 months prior to admission or during hospitalization. The patients underwent abdominal CT scans for multiple indications, including assessment of disease progression, monitoring of high-risk malignancies, and regular surveillance. Given the retrospective nature of this study, the Ethics Committee of Tianjin Medical University General Hospital (approval number: YX-136-01) approved a waiver of written informed consent, provided that strict measures were taken to protect patient privacy. However, for patients who were alive during the follow-up period, verbal informed consent was obtained via telephone to confirm their willingness to participate in the outcome assessment. All procedures were conducted in accordance with the Declaration of Helsinki and ethical guidelines for retrospective research in China. The de-identification process ensured that no personally identifiable information was included in the study dataset, and data access was restricted to the research team only.

### Estimation of the sample size

There is wide variation in the prevalence of distinct BC abnormalities owing to different definitions, diagnostic criteria, and assessment toolkits in the realm of hepatology (18). Therefore, the sample size of the current study was determined in terms of the investigated period rather than by calculation, that is, from January 2019 to December 2022.

### Body composition profiles annotated on computed tomography

A total of two well-trained practitioners, J.Y. and Q.L., read and annotated a single image located at the 3rd lumbar vertebra level for further assessment using a noncommercial processing software derived from MATLAB (Mathworks Inc., United States) (19). All radiologic examinations were carried out using a 64-row spiral CT scanner (Discovery 750 HD, GE Corp., United States). Broadly recognized Hounsfield unit (HU) thresholds were applied for skeletal muscle, SAT, and VAT, defined as −29 to +150 HU, −30 to −190 HU, and −50 to −150 HU, respectively. Notably, the number of BC features exhibited by each patient was recorded and analyzed to explore their potential negative impact on mortality, both in isolation and jointly.

### Other data retrieval

This study also collected a range of clinical and biochemical data for the recruited population, including age, sex, body mass index (BMI), etiologies of liver cirrhosis, related complications, and serum levels of bilirubin, creatinine, PT-INR, sodium, and albumin. In this respect, two scores were calculated to stratify the magnitude of underlying hepatic disease: the Child–Pugh score and the MELD-Sodium (MELD-Na) score.

### Outcome evaluation

The present study defined the primary outcome as 1-year all-cause mortality. The date of death was obtained by a well-trained practitioner, J.Y., during regular telephone follow-up, which continued until January 2024. Follow-up was censored at the date mentioned above or at the date of death if the event occurred earlier.

### Statistical analysis

Differences were compared using the Mann–Whitney U test and the chi-squared test or Fisher’s exact test for continuous (median [interquartile range]) and categorical variables (frequency [percentage]), respectively. Survival curves were generated using the Kaplan-Meier method, and differences were compared with the log-rank test. Univariate and multivariate Cox regression models (adjustment for albumin, the Child–Pugh score, and the MELD-Na score) were utilized to identify independent risk factors linked to the primary outcome. The results were reported as hazard ratios (HR) and 95% confidence intervals (CIs). Accordingly, a predictive nomogram for the female patients with decompensated cirrhosis was constructed and further verified by generating a calibration curve, a receiver operating characteristic (ROC) curve (area under the ROC curve [AUC]), and a decision curve. Statistical significance was defined as a two-sided *p*-value of <0.05, and all analyses were performed using R 3.3.2.^1^

## Results

### Patient characteristics

Table 1 shows the baseline demographic, clinical, and biochemical characteristics/features of the enrolled patients (*n* = 519). The median age was 64.0 years (57.0, 69.0), with a slight female predominance (51.6%). The etiology of cirrhosis was predominantly autoimmune/cholestasis (29.7%). The median MELD-Na score was 8.7 points (5.4, 11.7), and the majority of the patients were classified as Child–Pugh classification B (56.0%). The two most common liver cirrhosis-related complications were gastroesophageal variceal bleeding (69.7%) and ascites (61.3%). Furthermore, single BC abnormalities and overlapping features are also presented in Table 2.

**Table 1:** Baseline demographic, clinical, and biochemical characteristics of the study cohort.

Characteristic	Patients			
	All, *n* = 519 (100%)	Male, *n* = 251 (48.4%)	Female, *n* = 268 (51.6%)	*p*
Age (years)	64.0 (57.0, 69.0)	65.5 (58.0, 71.0)	62.0 (55.0, 67.0)	<0.001
BMI, kg/m^2^	23.8 (21.1, 27.3)	24.5 (22.9, 27.5)	23.4 (20.5, 26.7)	0.006
Etiology, *n* (%)				<0.001
HBV/HCV	124 (23.9)	65 (25.9)	59 (22.0)	
Alcohol	106 (20.4)	104 (41.4)	2 (0.7)	
Autoimmune/cholestasis	154 (29.7)	22 (8.8)	132 (49.3)	
MAFLD/other	135 (26.0)	60 (23.9)	75 (28.0)	
MELD score	8.4 (5.1, 11.2)	8.4 (4.3, 11.4)	8.5 (6.1, 11.0)	0.043
MELD-Na score	8.7 (5.4, 11.7)	8.9 (4.9, 12.6)	8.7 (6.4, 11.3)	0.910
AST (U/L)	31.0 (22.0, 49.0)	31.0 (21.0, 47.0)	31.5 (22.8, 50.3)	0.402
ALT (U/L)	23.0 (15.0, 36.0)	21.0 (15.0, 33.5)	23.0 (15.0, 37.0)	0.215
Total bilirubin (μmol/L)	21.0 (13.8, 37.0)	21.6 (13.7, 38.0)	21.0 (14.0, 36.5)	0.859
Albumin (g/L)	29.0 (26.0, 33.0)	28.0 (26.0, 33.0)	29.0 (25.0, 32.3)	0.657
Sodium (mmol/L)	140.0 (137.0, 142.0)	140.0 (137.0, 142.0)	140.0 (138.0, 142.0)	0.023
Creatinine (μmol/L)	58.0 (48.0, 72.0)	65.0 (55.5, 77.0)	52.0 (43.0, 62.0)	<0.001
PT-INR	1.3 (1.1, 1.4)	1.3 (1.2, 1.5)	1.23 (1.1, 1.4)	0.004
Complications, *n* (%)				
Ascites	318 (61.3)	157 (62.5)	161 (60.1)	0.625
Hepatic encephalopathy	43 (8.3)	24 (9.6)	19 (7.1)	0.389
GEVB	362 (69.7)	167 (66.5)	195 (72.8)	0.148
Infection	65 (12.5)	33 (13.1)	32 (11.9)	0.778
Child–Pugh score, *n* (%)				0.279
A	143 (27.6)	71 (28.3)	72 (26.9)	
B	291 (56.0)	133 (53.0)	158 (59.0)	
C	85 (16.4)	47 (18.7)	38 (14.1)	

BMI, body mass index; MELD, Model for End-Stage Liver Disease; MELD-Na, MELD-Sodium; AST, aspartate aminotransferase; ALT, alanine aminotransferase; MAFLD, metabolic dysfunction-associated fatty liver disease; PT-INR, prothrombin time-international normalized ratio; GEVB, gastroesophageal variceal bleeding.

**Table 2:** Single body composition abnormality and overlapping features.

Characteristic	Patients			
	All, *n* = 519 (100%)	Male, *n* = 251 (48.4%)	Female, *n* = 268 (51.6%)	*p*
VATI, cm^2^/m^2^	43.2 (29.0, 65.8)	36.3 (24.1, 52.8)	36.9 (24.5, 55.7)	0.069
High visceral adiposity, *n* (%)	291 (56.1)	188 (74.9)	103 (38.4)	<0.001
SATI, cm^2^/m^2^	43.2 (29.0, 65.8)	36.3 (24.1, 52.8)	52.8 (34.1, 76.4)	<0.001
Low subcutaneous adiposity, *n* (%)	124 (23.9)	88 (35.1)	36 (13.4)	<0.001
SMI, cm^2^/m^2^	44.8 (38.1, 51.9)	48.3 (41.3, 55.1)	41.7 (36.1, 48.0)	<0.001
Sarcopenia, *n* (%)	136 (26.2)	107 (42.6)	29 (9.7)	<0.001
IMAC	−0.55 (−0.70, −0.46)	−0.59 (−0.76, −0.48)	−0.53 (−0.66, −0.42)	<0.001
Myosteatosis, *n* (%)	79 (15.2)	35 (13.9)	44 (16.4)	0.508
Body composition features				<0.001
No features, *n* (%)	112 (21.6)	7 (2.8)	105 (39.2)	
1 feature, *n* (%)	229 (44.1)	112 (44.6)	117 (43.7)	
2 features, *n* (%)	138 (26.6)	95 (37.8)	43 (16.0)	
3 features, *n* (%)	35 (6.7)	32 (12.7)	3 (1.1)	
4 features, *n* (%)	5 (1.0)	5 (2.0)	0 (0.0)	

VATI, visceral adipose tissue index; SATI, subcutaneous adipose tissue index; SMI, skeletal muscle index; IMAC, intramuscular adipose tissue content.

### Body composition profiles stratified by sex

The prevalence of various combinations of BC abnormalities in the entire population is shown in Supplementary Figure S1. The most common isolated BC abnormality was high visceral adiposity (*n* = 170, 32.8%), and a small fraction of patients exhibited all four features simultaneously (*n* = 5, 1.0%) (Supplementary Figure S1A). Additionally, the most common combination of 2 features was sarcopenia with low subcutaneous adiposity (*n* = 50, 9.6%), while the most common combination of 3 features was sarcopenia, low subcutaneous adiposity, and high visceral adiposity (*n* = 20, 3.9%) (Supplementary Figure S1B).

**Figure 1:**
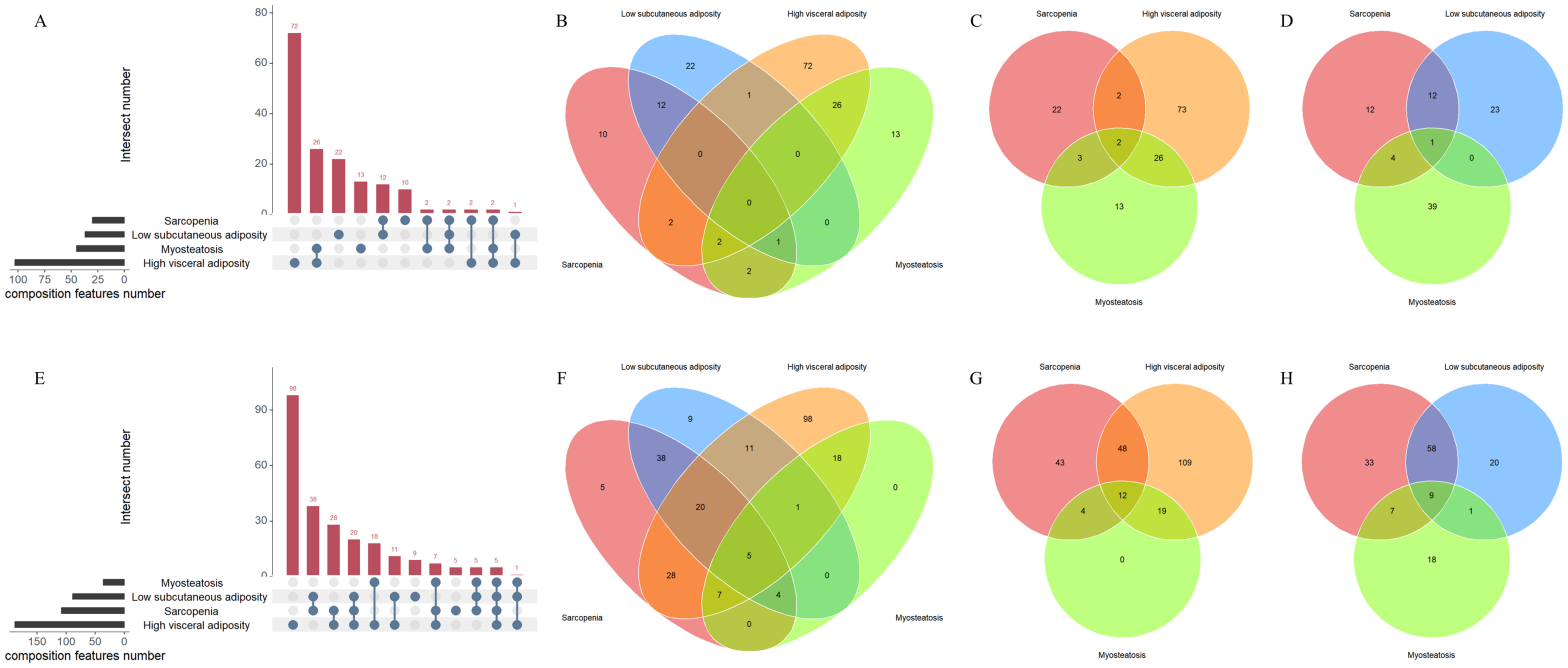
Upset plot and Venn diagram demonstrating distinct body composition profiles in the female **(A–D)** and male **(E–H)** patients with decompensated cirrhosis.

Given sex-related differences in hormone profiles, macronutrient metabolism, and physical activity, we aimed to explore the variations in BC profiles between male and female individuals in the context of decompensated cirrhosis (14). In the female individuals, the most common isolated BC abnormality was high visceral adiposity (*n* = 72, 26.9%), followed by low subcutaneous adiposity (*n* = 22, 8.2%) and myosteatosis (*n* = 13, 4.9%) (Figure 1A). Moreover, the most frequent two-feature combination was myosteatosis with high visceral adiposity, while the most frequent three-feature combination was sarcopenia, low subcutaneous adiposity, and myosteatosis (Figures 1B–D).

In the male individuals, the most common isolated BC abnormality was also high visceral adiposity (*n* = 98, 39.0%), followed by low subcutaneous adiposity (*n* = 9, 3.6%) and sarcopenia (*n* = 5, 2.0%) (Figure 1E). Furthermore, the most frequent two-feature combination was low subcutaneous adiposity with sarcopenia, while the most frequent three-feature combination was low subcutaneous adiposity, sarcopenia, and high visceral adiposity (Figures 1F–H). In total, five male patients exhibited all four features jointly (2.0%).

These results indicate distinct sex-specific patterns of BC abnormalities: The female patients demonstrated a predisposition toward adipose tissue disturbances, characterized by concurrent visceral adiposity and myosteatosis, whereas their male counterparts exhibited a higher tendency for musculoskeletal–adipose comorbidities, predominantly manifesting as sarcopenia with concomitant subcutaneous adipose depletion.

### Association between BC abnormalities and survival status

**Figure 2:**
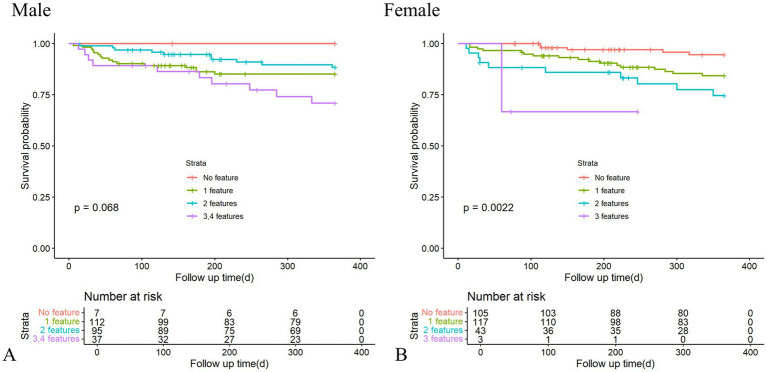
Kaplan–Meier curves showing 1-year all-cause mortality, with log-rank tests comparing different combinations of body composition features in the male **(A)** and female **(B)** patients with decompensated cirrhosis.

Figure 2 shows the association between different combinations of BC and survival status, stratified by sex, in the context of cirrhosis. In the female patients, a higher number of BC features was associated with an increased risk of 1-year all-cause mortality (log-rank *p* = 0.0022), while only a trend of marginal significance was observed in the male patients (log-rank *p* = 0.068). Univariate and multivariate Cox regression models, performed on a sex-specific basis, also showed that overlapping BC features were independently associated with worse survival status compared to no features in the female patients (2, 3 features: HR = 7.22, 95% CI 2.46, 21.25, *p* < 0.001; 1 feature: HR = 3.31, 95% CI 1.21, 9.07, *p* = 0.020). However, this association was not observed in the male patients with decompensated cirrhosis (Table 3; Supplementary Table S2).

**Table 3:** Univariate and multivariate Cox regression analyses in the female patients with decompensated cirrhosis.

	Univariate analysis		Multivariate analysis	
Variable	HR (95% CI)	*p*	HR (95% CI)	*p*
Age	1.02 (0.98, 1.05)	0.342		
BMI, kg/m^2^	0.93 (0.86, 1.01)	0.101		
Albumin (g/L)	0.87 (0.82, 0.93)	<0.001	0.87 (0.80, 0.95)	0.002
Total bilirubin (μmol/L)	1.01 (1.00, 1.01)	<0.001		
Sodium (mmol/L)	0.88 (0.83, 0.93)	<0.001		
Creatinine (μmol/L)	1.01 (1.01, 1.02)	<0.001		
MELD-Na	1.14 (1.08, 1.20)	<0.001	1.12 (1.06, 1.19)	0.001
SMI, cm^2^/m^2^	0.99 (0.95, 1.02)	0.444		
VATI, cm^2^/m^2^	1.00 (0.99, 1.02)	0.943		
SATI, cm^2^/m^2^	0.99 (0.98, 1.01)	0.418		
IMAC	4.98 (0.76, 32.73)	0.094		
Etiology				
HBV/HCV (*n* = 59)	1.00 (ref)			
Alcohol (*n* = 2)	4.43 (0.54, 36.05)	0.164		
Autoimmune/cholestasis (*n* = 132)	1.07 (0.44, 2.59)	0.886		
MAFLD/Other (*n* = 75)	1.03 (0.39, 2.78)	0.947		
Child–Pugh score				
A (*n* = 72)	1.00 (ref)			
B (*n* = 158)	2.06 (0.70, 6.09)	0.191	0.90 (0.27, 3.00)	0.860
C (*n* = 38)	6.16 (1.96, 19.37)	0.002	0.72 (0.16, 3.22)	0.671
Skeletal muscle status				
No sarcopenia (*n* = 239)	1.00 (ref)			
Sarcopenia (*n* = 29)	1.20 (0.42, 3.43)	0.728		
No Myosteatosis (*n* = 224)	1.00 (ref)			
Myosteatosis (*n* = 44)	2.45 (1.17, 5.15)	0.018		
No low subcutaneous adiposity (*n* = 232)	1.00 (ref)			
Low subcutaneous adiposity (*n* = 36)	2.68 (1.24, 5.76)	0.012		
No high visceral adiposity (*n* = 165)	1.00 (ref)			
High visceral adiposity (*n* = 103)	1.74 (0.88, 3.44)	0.113		
Body composition features-number				
No features (*n* = 105)	1.00 (ref)			
1 feature (*n* = 117)	3.12 (1.15 8.47)	0.025	3.31 (1.21, 9.07)	0.020
2, 3 features (*n* = 46)	5.75 (2.00, 16.55)	0.001	7.22 (2.46, 21.25)	<0.001

VATI, visceral adipose tissue index; SATI, subcutaneous adipose tissue index; SMI, skeletal muscle index; IMAC, intramuscular adipose tissue content; BMI, body mass index; MELD-Na, Model for End-Stage Liver Disease -Sodium; HBV, hepatitis B virus; HCV, hepatitis C virus; MAFLD, metabolic dysfunction-associated fatty liver disease.

### Construction of a predictive nomogram and relevant performance

**Figure 3:**
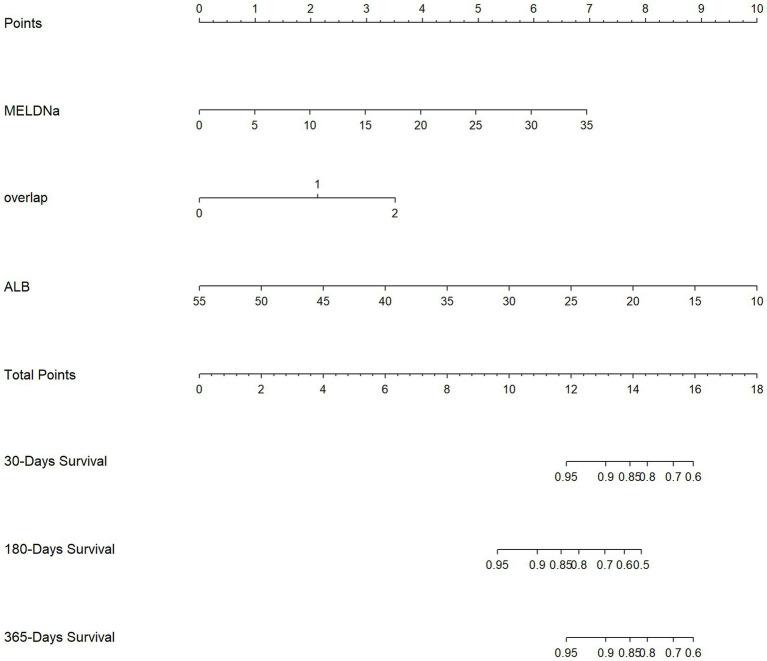
Nomogram incorporating overlapping body composition abnormalities in relation to 1-year survival status among the female patients with decompensated cirrhosis. MELD-Na, model for end-stage liver disease-sodium score; ALB, albumin. A step-by-step usage example: (1) For a given female patient, locate her values for each variable on the corresponding nomogram axis (e.g., Albumin = 28 g/L, MELD-Na = 15, number of BC abnormalities = 2). (2) Draw a vertical line from each value to the “Points” axis to determine the individual score for each parameter. (3) Sum all the assigned points to obtain the “Total Points.” (4) Locate this “Total Points” value on the respective axis. (5) Draw a vertical line downward from the “Total Points” axis to the “1-Year Survival Probability” axis to determine the patient’s predicted 1-year survival probability.

A nomogram incorporating overlapping BC features and other significant prognostic factors was constructed (Figure 3). The C-index of the proposed model was 0.802 (95% CI 0.731, 0.873), and it yielded an AUC of 0.809 (Supplementary Figure S2A). In addition, time-dependent AUC analysis revealed consistently good discrimination for 1-year all-cause mortality compared to the conventional MELD-Na score (Supplementary Figure S2B). The decision and calibration curves demonstrated the practical utility of our model, as well as a good correlation between the predicted and observed survival (Supplementary Figures S2C–F).

## Discussion

To the best of our knowledge, this is the first study to investigate CT-defined BC profiles and, in particular, their combined impact on long-term outcomes while accounting for sex differences. Our main findings indicate a significant sex-specific variability in BC abnormalities. Notably, the presence of overlapping BC features was associated with an increased risk of mortality in the female patients, but not the male patients, with decompensated cirrhosis. The improved prognostic performance of our newly developed nomogram, compared to the conventional MELD-Na scoring system, underscores its clinical significance and practical relevance by incorporating distinct BC phenotypes to enhance prognostic accuracy. Future studies are warranted to understand the integrated metabolic pathways between subcutaneous adipose tissue, visceral adipose tissue, muscle, and liver in the context of advanced chronic liver disease.

Most research focuses on a single BC abnormality in patients with various stages of cirrhosis, inevitably leaving knowledge gaps. To date, only one study has reported unpublished data examining overlapping BC phenotypes in a cohort of 645 patients (female: 208, male: 437) eligible for liver transplantation (16). In this regard, Ebadi et al. reported that myosteatosis was the most prevalent single BC feature (*n* = 340, 53%), and this pattern was consistent across both sexes. Furthermore, myosteatosis frequently coexisted with low subcutaneous adiposity (*n* = 73, 35%) and sarcopenia (*n* = 114, 26%) in female and male individuals, respectively. These findings differ markedly from our observations of BC profiles among Chinese patients admitted with decompensating insults (Figure 1). Several factors should be considered when evaluating the influence of ethnicity on BC, particularly between Western and Asian individuals, including, but not limited to, dietary habits, body size, racial characteristics, and quality of life (16). The potential mechanisms include early-life appearance, environmental exposures, hormone levels, and genetic control, all of which may influence the development and maintenance of bone, skeletal muscle, and fat mass (20).

Another important finding is the identification of sex-specific differences in overlapping BC features. In the current study, we found that the female patients with decompensated cirrhosis were more likely to exhibit adipose tissue disturbances, while the male patients were more likely to show muscular alterations. One possible interpretation is that the metabolic pathway linked to muscle wasting in male individuals with cirrhosis resembles that observed in critical illness. In contrast, the catabolic pattern of fat loss in female counterparts mimics the situation encountered in starvation or chronic diseases (21). In particular, decreased glucose oxidation combined with increased fatty acid availability and utilization under conditions of high-energy demand may explain the relatively lower muscle degradation and greater metabolic efficiency observed in female patients with cirrhosis (15).

Although the association between specific isolated BC abnormalities, such as sarcopenia or high visceral adiposity, and 1-year all-cause mortality was not significant in unadjusted Cox regression, our previous report confirmed their predictive value for 3-year mortality (8). This difference may be attributable to the relatively smaller cohort in the earlier study and the different endpoints examined. In contrast, the prognostic significance of myosteatosis and low visceral adiposity was confirmed in our univariate Cox model. Similarly, a study involving 372 cirrhotic patients in China revealed that both excessively low and high SAT were associated with poorer outcomes, while a moderate amount of SAT appeared beneficial for long-term survival (22). Most recently, myosteatosis, either single or combined with sarcopenia, was identified as the most frequent form of muscle impairment and exerted a major prognostic impact on the course of liver disease (23). Notably, the aforementioned study also failed to identify an independent predictive role for isolated sarcopenia. Body habitus parameters are interrelated and multifaceted (24), suggesting that reducing an individual BC feature to simple binary categories may be an oversimplification. Notably, among female patients, worsening survival status, as estimated by the Kaplan-Meier curve, was correlated with a higher number of adverse features.

Our findings also revealed a critical sex-based disparity: The female patients with more than two BC abnormalities experienced significantly reduced survival, whereas the male patients with a comparable BC burden showed only marginal prognostic deterioration (*p* = 0.069). This suggests that fewer BC abnormalities in female individuals may trigger disproportionately severe outcomes. We propose two underlying mechanisms: First, sex-specific metabolic adaptations in cirrhosis may play a crucial role. Female individuals predominantly depend on fatty acid oxidation, whereas male individuals rely more on protein catabolism (15). Consequently, adipose tissue abnormalities in female individuals may disrupt energy homeostasis more profoundly. Second, female individuals may possess a lower threshold for BC abnormality impact. Given their physiological predisposition toward higher SAT and lower muscle mass (14), even moderate BC alterations could more substantially compromise nutritional and immune reserves. This “lower tolerance threshold” concept reinforces the need for sex-specific BC criteria in prognostic evaluation. The distinct combination patterns further suggest that a “one-size-fits-all” approach to body composition interventions is inappropriate. Instead, personalized strategies based on sex and BC profiles are needed.

A major strength of our study is the successful development of a predictive nomogram incorporating both traditional scoring systems/measures (i.e., MELD-Na and albumin) and varying degrees of BC abnormalities. This newly proposed model exhibits strong discriminatory performance, good calibrated capabilities, and incremental clinical utility. It enables early identification of high-risk patients who might be overlooked by traditional scoring systems, particularly female individuals with specific body composition phenotypes. This allows for targeted interventions, such as nutritional optimization and physical therapy, at earlier disease stages. Indeed, it has been argued that BC assessments using CT imaging, which is known as non-invasive and objective, should be adopted for prognostication by leading experts and reputable institutions (16, 18). We acknowledge that further validation is needed before widespread implementation. Our proposed pathway includes the following: (1) external validation in multicenter cohorts with diverse ethnic backgrounds, (2) assessment of clinical utility through randomized trials evaluating nomogram-guided management, and (3) standardization of body composition measurement protocols across different CT platforms.

There are several limitations that should be acknowledged. First, the sample size was determined by the study period rather than through a formal power calculation. Second, we did not include a nutritional assessment due to the retrospective design of the study. Third, we applied outcome-based cutoffs to define various BC abnormalities, as there is currently a lack of unanimous definitions and standardized diagnostic criteria for sarcopenia, myosteatosis, and adiposity. Finally, we were unable to show a negative impact of overlapping BC features in the male patients. Taken together, future prospective studies involving larger cohorts are warranted to clarify the association between complex BC profiles and their influence on prognostication in the context of cirrhosis.

## Conclusion

In conclusion, wide variation in BC profiles, with sex-based differences, was observed in the context of decompensated cirrhosis. The number of BC features was associated with worse survival in the female patients, highlighting the need for tailored treatment that considers the multifaceted nature of human BC. Future studies are urgently needed to clarify the integrated metabolic pathways between muscle, distinct adipose tissues, and the liver in patients with chronic end-stage liver diseases.

## Data Availability

The raw data supporting the conclusions of this article will be made available by the authors, without undue reservation.

## Footnotes

1
http://www.R-project.org

## References

[1] Dq H Na T Tacke F Ll G Arrese M Bugianesi E . Global epidemiology of cirrhosis—aetiology, trends and predictions. Nat Rev Gastroenterol Hepatol. (2023) 20:388–98. doi: 10.1038/s41575-023-00759-2, 36977794 PMC10043867

[2] Arnold J Avila E Idalsoaga F Diaz L Ayala Valverde M Ayares G . Advances in the diagnosis and management of hepatorenal syndrome: insights into Hrs-Aki and liver transplantation. eGastroenterology. (2023) 1:e100009. doi: 10.1136/egastro-2023-100009, 39943997 PMC11770447

[3] Singh J Ebaid M Saab S. Advances in the management of complications from cirrhosis. Gastroenterol Rep. (2024) 12:goae072. doi: 10.1093/gastro/goae072, 39104730 PMC11299547

[4] Godfrey E Malik T Lai J Mindikoglu A Galvan N Cotton R . The decreasing predictive power of meld in an era of changing etiology of liver disease. Am J Transplant. (2019) 19:3299–307. doi: 10.1111/ajt.15559 31394020

[5] Guo G Yang W Li J Yang Z Liang J Sun C. The development and appraisal of meld 3.0 in liver diseases: good things never come easy. J Clin Transl Hepatol. (2025) 13:62–8. doi: 10.14218/Jcth.2024.00303, 39801783 PMC11712091

[6] Wang S Guo G Wang H Zhang X Yang W Yang J . Improved discrimination and predictive ability of novel prognostic scores for long-term mortality in hospitalized patients with cirrhosis. J Clin Transl Hepatol. (2025) 13:484–92. doi: 10.14218/jcth.2025.00004, 40474888 PMC12134911

[7] Wang H Zhao T Guo G Yang W Zhang X Yang F . Global leadership initiative on malnutrition-defined malnutrition coexisting with visceral adiposity predicted worse long-term all-cause mortality among inpatients with decompensated cirrhosis. Nutr Diabetes. (2024) 14:76. doi: 10.1038/s41387-024-00336-9, 39333477 PMC11436742

[8] Hou L Deng Y Fan X Zhao T Cui B Lin L . A sex-stratified prognostic nomogram incorporating body compositions for long-term mortality in cirrhosis. JPEN J Parenter Enteral Nutr. (2021) 45:403–13. doi: 10.1002/jpen.1841, 32359094

[9] Yang J Liu Q Sun C. Quality and quantity? The clinical significance of myosteatosis in various liver diseases: a narrative review. J Clin Transl Hepatol. (2025) 13:1092–106. doi: 10.14218/jcth.2025.00383 41473262 PMC12745355

[10] Zamboni M Mazzali G Brunelli A Saatchi T Urbani S Giani A . The role of crosstalk between adipose cells and myocytes in the pathogenesis of sarcopenic obesity in the elderly. Cells. (2022) 11:3361. doi: 10.3390/cells11213361, 36359757 PMC9655977

[11] Hui Y Cui B Wang X Sun M Li Y Yang W . Sarcopenic obesity in liver disease: handling both sides of the penny. Portal Hypertens Cirrhosis. (2022) 1:42–56. doi: 10.1002/poh2.10

[12] Mao L Li C Wang X Sun M Li Y Yu Z . Dissecting the contributing role of divergent adipose tissue to multidimensional frailty in cirrhosis. J Clin Transl Hepatol. (2023) 11:58–66. doi: 10.14218/Jcth.2022.00027, 36406322 PMC9647104

[13] Liu Q Liu J Sun C. Clinical significance and therapeutic approach concerning various abdominal adipose tissue irregularities in end-stage liver disease. Obes Rev. (2025) 26:e13955. doi: 10.1111/obr.13955, 40485111

[14] Bredella M. Sex differences in body composition. Adv Exp Med Biol. (2017) 1043:9–27. doi: 10.1007/978-3-319-70178-3_2 29224088

[15] Lundsgaard A Kiens B. Gender differences in skeletal muscle substrate metabolism—molecular mechanisms and insulin sensitivity. Front Endocrinol (Lausanne). (2014) 5:195. doi: 10.3389/fendo.2014.00195, 25431568 PMC4230199

[16] Ebadi M Bhanji R Tandon P Mazurak V Baracos V Montano-Loza A. Review article: prognostic significance of body composition abnormalities in patients with cirrhosis. Aliment Pharmacol Ther. (2020) 52:600–18. doi: 10.1111/apt.15927 32621329

[17] Wang H Wang S Li C Yang W Guo G Hui Y . Coexistent glim-defined malnutrition and sarcopenia increase the long-term mortality risk in hospitalized patients with decompensated cirrhosis. Ann Nutr Metab. (2023) 79:423–33. doi: 10.1159/000534152, 37725950

[18] Lai J Tandon P Bernal W Tapper E Ekong U Dasarathy S . Malnutrition, frailty, and sarcopenia in patients with cirrhosis: 2021 practice guidance by the American Association for the Study of Liver Diseases. Hepatology. (2021) 74:1611–44. doi: 10.1002/hep.32049, 34233031 PMC9134787

[19] Kim S Kim J Jeong W Lee J Kim Y Choi D . Semiautomatic software for measurement of abdominal muscle and adipose areas using computed tomography: a strobe-compliant article. Medicine (Baltimore). (2019) 98:e15867. doi: 10.1097/Md.0000000000015867, 31145342 PMC6708812

[20] Heymsfield S Peterson C Thomas D Heo M Schuna J Jr. Why are there race/ethnic differences in adult body mass index-adiposity relationships? A quantitative critical review. Obes Rev. (2016) 17:262–75. doi: 10.1111/obr.12358, 26663309 PMC4968570

[21] Harimoto N Yoshizumi T Shimokawa M Sakata K Kimura K Itoh S . Sarcopenia is a poor prognostic factor following hepatic resection in patients aged 70 years and older with hepatocellular carcinoma. Hepatol Res. (2016) 46:1247–55. doi: 10.1111/hepr.12674, 26880049

[22] Zhu M Li H Yin Y Ding M Ca P Fg R . U-shaped relationship between subcutaneous adipose tissue index and mortality in liver cirrhosis. J Cachexia Sarcopenia Muscle. (2023) 14:508–16. doi: 10.1002/jcsm.13154, 36577511 PMC9891908

[23] Di Cola S D'Amico G Caraceni P Schepis F Loredana S Lampertico P . Myosteatosis is closely associated with sarcopenia and significantly worse outcomes in patients with cirrhosis. J Hepatol. (2024) 81:641–50. doi: 10.1016/j.jhep.2024.05.020, 38782120

[24] Boshier PR Heneghan R Markar SR Baracos VE Low DE. Assessment of body composition and sarcopenia in patients with esophageal cancer: a systematic review and meta-analysis. Dis Esophagus. (2018) 31:doy047. doi: 10.1093/dote/doy047, 29846548

