# Supplementary material for: Overlap between body composition abnormalities and sex-specific prognostication in decompensated cirrhosis
Source: Front Nutr. 2026 Jan 13;12:1705226. doi: 10.3389/fnut.2025.1705226 (PMC12834759; doi:10.3389/fnut.2025.1705226)
Supplement: Supplementary file 1 [file Table_1.DOCX]

**Table S1** Body composition abnormality and related cutoffs in the current study

| Body composition abnormalities | Area and definition | Interpretation | Outcome-based cutoffs | |
| --- | --- | --- | --- | --- |
|  |  |  | Females | Males |
| IMAC | L3 region of the interest (ROI) of the multifidus muscle/(ROI) of subcutaneous adipose | Increased values imply more low attenuation myosteatotic muscle, thus lower muscle quality | > -0.37 | > -0.44 |
| SMI (cm^2^/m^2^) | L3 whole skeletal muscle area/squared height | Decreased values imply wasting muscle mass, thus lower muscle quantity | < 32.46 | < 46.96 |
| VATI (cm^2^/m^2^) | L3 whole visceral adipose tissue area/squared height | Increased values imply excessive accumulation of adipose tissue in the visceral depot, thus high visceral adiposity | > 44.02 | > 28.42 |
| SATI (cm^2^/m^2^) | L3 whole subcutaneous adipose tissue area/squared height | Decreased values imply insufficient storage of adipose tissue in the subcutaneous depot, thus low subcutaneous adiposity | < 26.75 | < 29.1 |

IMAC, intramuscular adipose tissue content; SMI, skeletal muscle index; VATI, visceral adipose tissue index; SATI, subcutaneous adipose tissue index.

We applied the following attenuation cutoffs to discriminate between various tissue components on cross-sectional CT images, aligning with the literature definitions: Skeletal muscle: -29 to 150 HU, visceral adipose tissue: -150 to -50 HU, and subcutaneous adipose tissue: -190 to -30 HU. The sex-specific cutoffs were derived from our prior report concerning the survival status of 3-year all-cause mortality in the context of decompensated cirrhosis.
