# Supplementary material for: Overlap between body composition abnormalities and sex-specific prognostication in decompensated cirrhosis
Source: Front Nutr. 2026 Jan 13;12:1705226. doi: 10.3389/fnut.2025.1705226 (PMC12834759; doi:10.3389/fnut.2025.1705226)
Supplement: Supplementary file 2 [file Table_2.DOCX]

**Table S2** Univariate and Multivariate Cox analysis in male patients with decompensated cirrhosis.

|  | Univariate analysis | | Multivariate analysis | | |
| --- | --- | --- | --- | --- | --- |
| Variable | HR (95% CI) | p | HR (95% CI) | p | |
| age | 1.02 (0.99, 1.06) | 0.184 |  |  | |
| BMI, kg/m^2^ | 0.97 (0.90, 1.05) | 0.436 |  |  | |
| MELD-Na | 1.09 (1.04, 1.14) | <0.001 | 1.08 (1.02, 1.14) | 0.013 | |
| Albumin (g/L) | 0.86 (0.79, 0.93) | <0.001 | 0.91 (0.83, 0.99) | 0.037 | |
| Total bilirubin (µmol/L) | 1.00 (1.00, 1.01) | 0.040 |  |  | |
| Sodium (mmol/L) | 0.94 (0.91, 0.98) | 0.003 |  |  | |
| Creatinine (µmol/L) | 1.00 (1.00, 1.01) | 0.003 |  |  | |
| SMI, cm^2^/m^2^ | 0.99 (0.95, 1.02) | 0.361 |  |  | |
| VATI, cm^2^/m^2^ | 1.00 (0.99, 1.02) | 0.526 |  |  | |
| SATI, cm^2^/m^2^ | 0.99 (0.97, 1.00) | 0.155 |  |  | |
| IMAC | 0.99 (0.81, 1.20) | 0.906 |  |  | |
| Etiology |  |  |  |  | |
| HBV/HCV (n = 65) | 1.00 (ref) |  |  |  | |
| Alcohol (n = 104) | 1.51 (0.66, 3.43) | 0.325 |  |  | |
| Autoimmune/Cholestasis (n = 22) | 0.33 (0.04, 2.62) | 0.293 |  |  | |
| MAFLD/Other (n = 60) | 0.90 (0.33, 2.47) | 0.834 |  | |  |
| CTP classification |  |  |  |  | |
| A (n = 71) | 1.00 (ref) |  |  |  | |
| B (n = 133) | 3.15 (1.09, 9.12) | 0.034 | 2.08 (0.43, 10.09) | 0.363 | |
| C (n = 47) | 3.62 (1.12, 11.76) | 0.032 | 1.16 (0.19, 6.86) | 0.874 | |
| **Skeletal muscle status** |  |  |  |  | |
| No sarcopenia (n = 144) | 1.00 (ref) |  |  |  | |
| Sarcopenia (n = 107) | 1.57 (0.82, 3.02) | 0.177 |  |  | |
| No Myosteatosis (n = 216) | 1.00 (ref) |  |  |  | |
| Myosteatosis (n = 35) | 1.53 (0.67, 3.48) | 0.316 |  |  | |
| No low subcutaneous adiposity (n = 163) | 1.00 (ref) |  |  |  | |
| Low subcutaneous adiposity (n = 88) | 1.18 (0.60, 2.31) | 0.626 |  |  | |
| No high visceral adiposity (n = 63) | 1.00 (ref) |  |  |  | |
| High visceral adiposity (n = 188) | 1.38 (0.60, 3.15) | 0.445 |  |  | |
| **Body composition features-number** |  |  |  |  | |
| No,1 feature (n = 119) | 1.00 (ref) |  |  |  | |
| 2 features (n = 95) | 0.75 (0.34, 1.65) | 0.471 | 0.62 (0.27, 1.41) | 0.252 | |
| 3, 4 features (n = 37) | 2.08 (0.95, 4.59) | 0.069 | 1.10 (0.46, 2.66) | 0.831 | |

VATI, visceral adipose tissue index; SATI, subcutaneous adipose tissue index; SMI, skeletal muscle index; IMAC, intramuscular adipose tissue content; BMI, body mass index; MELD-Na, model for end-stage liver disease-Sodium; HBV, hepatitis B virus; HCV, hepatitis C virus; MAFLD, metabolic dysfunction-associated fatty liver disease.
