# Supplementary figures and images for: Overlap between body composition abnormalities and sex-specific prognostication in decompensated cirrhosis
Source: Front Nutr. 2026 Jan 13;12:1705226. doi: 10.3389/fnut.2025.1705226 (PMC12834759; doi:10.3389/fnut.2025.1705226)

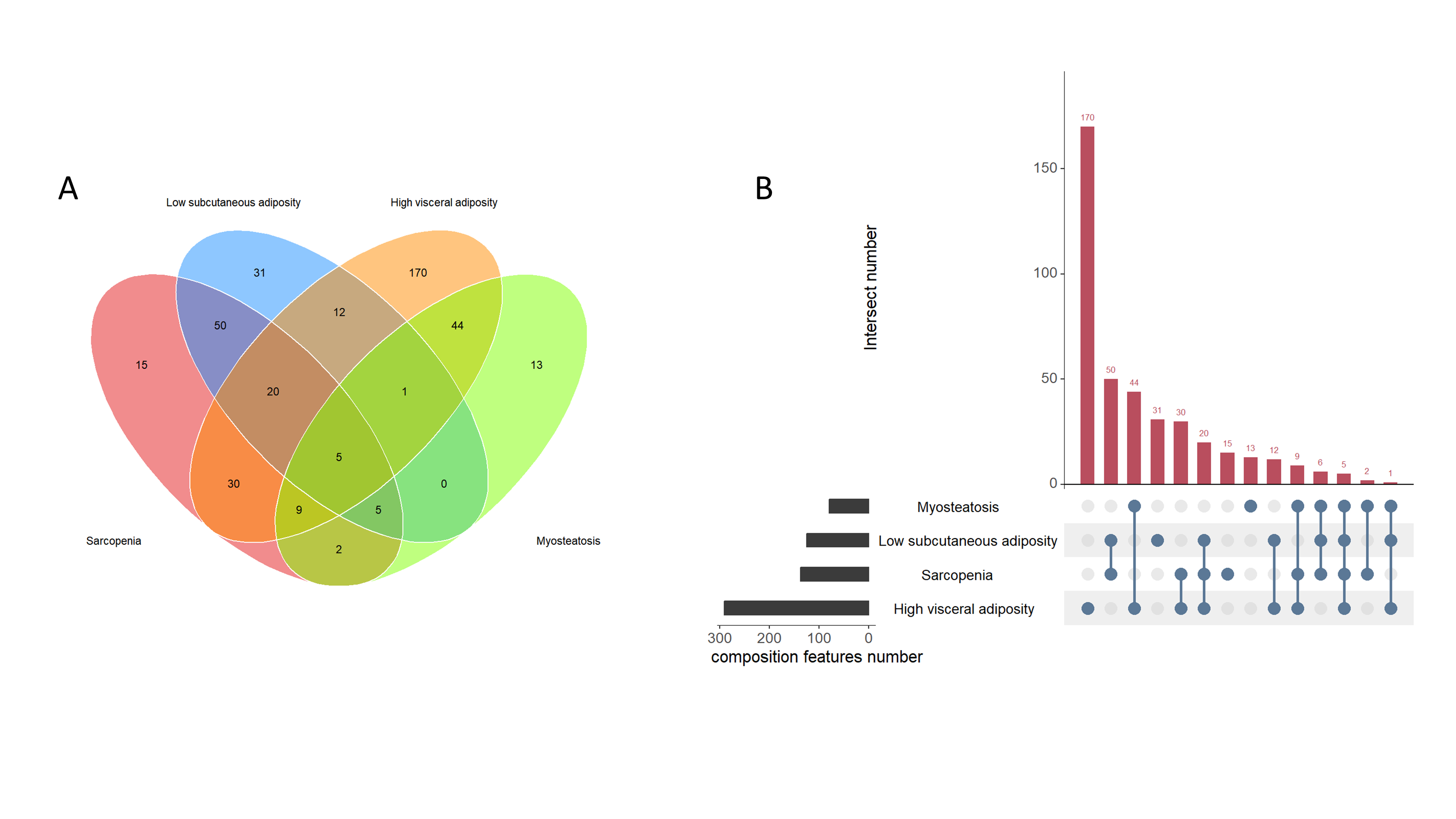

Supplement: Supplementary file 3 [file Image_1.TIF]

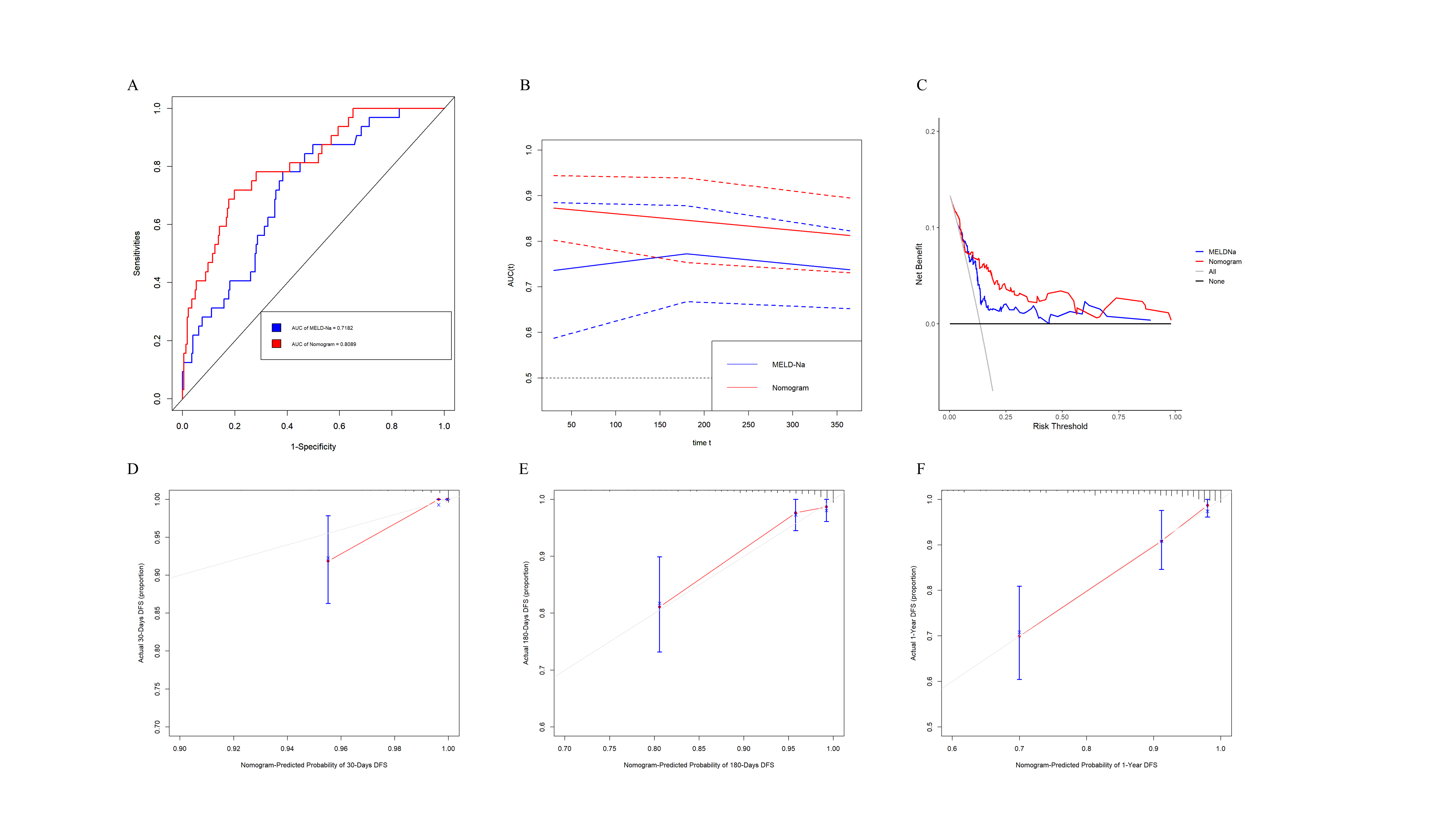

Supplement: Supplementary file 4 [file Image_2.TIF]
